# Synthesis of double-shelled periodic mesoporous organosilica nanospheres/MIL-88A-Fe composite and its elevated performance for Pb^2+^ removal in water

**DOI:** 10.1038/s41598-023-35149-w

**Published:** 2023-05-19

**Authors:** Sara S. E. Ghodsinia, Hossein Eshghi, Arezou Mohammadinezhad

**Affiliations:** Department of Chemistry, Faculty of Science, Ferdowsi University of Mashhad, Mashhad, 9177948974 Iran

**Keywords:** Catalysis, Environmental chemistry, Green chemistry, Organic chemistry

## Abstract

Herein, we report the synthesis of double-shelled periodic mesoporous organosilica nanospheres/MIL-88A-Fe (DSS/MIL-88A-Fe) composite through a hydrothermal method. To survey the structural and compositional features of the synthesized composite, a variety of spectroscopic and microscopic techniques, including FT-IR, XRD, BET, TEM, FE-SEM, EDX, and EDX-mapping, have been employed. A noteworthy point in this synthesis procedure is the integration of MOF with PMO to increase the adsorbent performance, such as higher specific surface area and more active sites. This combination leads to achieving a structure with an average size of 280 nm and 1.1 μm long attributed to DSS and MOF, respectively, microporous structure and relatively large specific surface area (312.87 m^2^/g). The as-prepared composite could be used as an effective adsorbent with a high adsorption capacity (250 mg/g) and quick adsorption time (30 min) for the removal of Pb^2+^ from water. Importantly, DSS/MIL-88A-Fe composite revealed acceptable recycling and stability, since the performance in Pb^2+^ removal from water remained above 70% even after 4 consecutive cycles.

## Introduction

For all human activities, safe water is an essential material, but the presence of pollutants is one of the most significant human challenges to obtaining clean water. The rapid development of industrialization leads to increased heavy metal pollution in the environment^1–3^. To reduce water pollution from the discharge of heavy metals into nature, the environmental protection agency (EPA) has set specified allowed limits for this purpose. The maximum acceptable concentration of Pb^2+^ in industrial discharge and drinking water according to the guidelines of WHO and the EPA was determined to approach 0.01 and 0.015 mg/L, respectively^4,5^. This value for Pb (II) in wastewater is 0.05 mg/L, based on the EPA guidance^6,7^. Furthermore, lead-ion concentrations in industrial wastewaters are about 200–500 mg/L. It should be noted that this value is remarkably higher than the standard quality of water, hence, before discharging the wastewater to waterways or sewage systems, the lead-ion concentration must be reduced to a level of 0.05–0.10 mg/L^6,8,9^. Heavy metal ions including lead, hurt human health and the environment. These heavy metals can lead to many diseases and complications in the body^10,11^. Therefore, removing heavy metals, including Pb^2+^, from water and wastewater is of great importance not only for protecting water resources but also for the permanent survival of humans. According to this essential issue, scientists have focused on new technologies that will enable heavy metals to be eliminated from environmental supplies^12–15^. Generally, some treatment methods utilized to purify water by entrapment of heavy metal and radioactive ions have been focused on adsorption-^16^, membrane-^17^, chemical-^18^, electric-^18^, and photocatalytic-^19,20^ based treatments. Among them, the adsorption process is one of the most efficient methods to deal with heavy metal ions because of its simple performance, various sources of adsorbent, affordable cost, simple operation, high efficiency, and regenerative ability of adsorbents^21^. In recent years, conventional adsorbent materials including, metal oxides^22–27^, active carbon^28–34^, and carbon nanotubes^35–40^ have gained enormous attention. Undoubtedly these materials represent great adsorbent behaviour; nevertheless, some of them still suffer from some disadvantages including, small sizes and pore volumes, down adsorption kinetics, elaborate preparation, difficult renewal, and low adsorption efficiency. Hence, there is an urgent need to develop new adsorption materials.

Recently, porous materials such as metal–organic frameworks (MOF)^41–46^, and hollow periodic mesoporous organosilica (PMOs)^47–56^ are attracted an inclusive application outlook in the field of adsorption. Metal–organic frameworks (MOF), which are also known as a new class of hybrid and crystalline materials in the field of metal–organic materials (MOMs)^57–59^ are constructed by metal centers or clusters that bridged through strong coordination bonds with organic linkers^60–64^. The synthesis procedure for obtaining MOF NPs is classified into two major categories, including hydrothermal^65^ and solvothermal techniques^66,67^. In both procedures, two solutions containing the metal ions in their stable oxidation states, i.e., alkaline, alkaline earth, transition metal, and rare earth elements^68^ were mixed with the organic linkers such as poly-carboxylic molecules and poly-azaheterocycles^69^ to achieve a wide range of crystalline and stable MOFs structures. In recent years, these materials have experienced rapid and extensive growth of attention owing to their attractive features such as high surface area^70^, well-defined pore architectures^71^, and tunable structural features^72^. Their unique properties make them a great candidate for many applications, including gas storage^73^, purification^74^, molecular sensing^75^, drug delivery^76^, organic catalysts, and water purification^77^. In the case of water treatment applications, using MOFs with small-sized powder is associated with some risks due to the high affinity of these compounds toward the water which leads to increase the possibility of agglomeration and therefore difficult recovery^45^. As a result of this problem, MOF nanoparticles can directly enter drinking water^78^, and cause long-term environmental nano-toxicity, heavy metal pollution problems, and affect human health^79^. On the other hand, some of the MOF structures are sensitive to moisture and water which leads to a structural collapse in metal–organic frameworks (MOFs), which is a serious disadvantage in applied usage^80^. The main reason for this phenomenon can be explained by the structure of MOFs (the nature of the linker and the metal cluster) and the activation process which leads to the bridging effect that occurs in water adsorption^46^. For example, in the activation process of HKUST-1 MOF (= (Cu_3_(BTC)_2_) (BTC = benzene-1,3,5-tricarboxylate)) which is composed of copper ions capped by axial water ligand and BTC linkers, the axial water ligands were removed, resulting a new arrangement in the geometry of the cupric center toward a relatively stable square planar coordination^81^. However, according to the reported articles^46,81^, HKUST-1 has a high water adsorption affinity and showed no long-term stability when directly contacting with water. Against this group, some of MOF materials, such as MIL-101(Fe), exhibit excellent water stability. They can introduce as great candidates for promising materials for water adsorption applications such as the removal of heavy metals^82–85^. In order to increase the advantages of MOF compounds, incorporating excellent adsorbents such as PMOs with these materials leads to forming a composite with enhanced mechanical properties.

So-called periodic mesoporous organosilica (PMOs) as advanced hybrid organic–inorganic materials have been investigated with great interest owing to their unique physicochemical properties such as porous channels^54^, robust porous organic–inorganic framework^86^, adjustable pore size organization^87,88^, biocompatibility^89^, and the highest organic content in the (nano)material^90^. These excellent properties render PMOs attractive for applications in many fields, such as adsorption^47–52^, catalysis^91–97^, light-harvesting^98,99^, electronics^100–102^, drug release studies in simulated biological media^103–108^, chromatography^109^, enzyme immobilization^110,111^, and bactericides^112,113^. PMOs have a lot of applications as catalysts in many fields such as the synthesis of dihydropyrano[3,2-c] chromene derivatives^114^, sonogashira reaction^115^, Chan-Lum coupling^116^, condensation of a variety of different aldehydes with malononitrile^117^, Knoevenagel condensation^118^, oxidation of alcohols^119,120^, clean production of polyhydroquinolines^121^, and Heck reaction^122^. Also, the application of these materials as adsorbents for selective adsorption and separation of heavy metals (like lead) is considered one of the most attractive research hotspots. It is interesting to note that the removal of heavy metal ions using PMOs is sensitive to functional silane precursors in their frameworks^123^. In the other words, according to the basis of the research purpose and the type of metal ions to be removed, the PMO precursors are changed and selected very carefully (according to the hard-soft acid–base (HSAB) theory). In this regard, hard acids (such as Mg^2+^, Ca^2+^, and Cr^3+^) have an affinity for hard bases with high electronegativity; soft acids (such as Hg^2+^, Pd^2+^, and Hg^2+^) react with soft bases and have a powerful affinity to soft *N* and *O*-donor ligands and boundary acids (such as Zn^2+^, Fe^2+^, and Cr^2+^) react with boundary bases^124–126^. PMOs which comprised siloxane units bridged by organic groups, are traditionally synthesized through both sol–gel and grafting methods^119^. The sol–gel process involves mixing the organo-bridged alkoxysilanes (RʹO)_3_Si–R–Si(ORʹ)_3_ with copolymers or surfactants as a blocking agent^56,127,128^. While, in the grafting method, a non-bridged organosilica precursor was attached to a pre-prepared PMO material^119^. By inserting some specific organic groups into the pore walls of PMOs, new silica (nano) materials with both advantages of the organic and inorganic units were obtained^56,87,127–135^. Interestingly, by modifying the internal and external surface of PMOs, the hydrophilicity and hydrophobicity of the pores adjust, resulting in to control the properties of these materials in solvents^136–138^. Moreover, according to the reported literature^139^, under certain conditions such as pH, redox, photochemical, or biochemical conditions, the PMO materials may changes. Hereupon, modification of these compounds with other materials (such as MOFs) can be a great solution to solve their problems.

In this work, we focused on synthesizing a new nanocomposite through the integration of double-shelled periodic mesoporous organosilica (DSS) with MIL-88A-Fe aimed at enhancing water stability and adsorption capacity. In the following, the performance of DSS/MIL-88A-Fe as adsorbents will be examined to eliminate of lead from water. It provides a theoretical basis for integration of PMO with MOFs in the treatment of heavy metal ions in water.

## Experimental section

The FTIR spectra were recorded in transmission mode 4000–400 cm^−1^ on Thermo Nicolet Avatar 370 spectrometer equipped at room temperature. X-ray powder diffraction (XRD) was performed on a PANalytical Company X’Pert Pro MPD diffractometer with Cu Kα (λ = 0.154 nm) radiation. Nitrogen adsorption isotherms were measured on a Quantachrome Instruments version 2.2 using N_2_ as the adsorbate at − 196 °C. Transmission electron microscopy (TEM) was carried out using an EM10C-100 kV microscope (ZEISS Company). FE-SEM images, EDX, and EDX-mapping were recorded by TESCAN (model: Sigma VP) scanning electron microscope operating at a low accelerating voltage of 15.00 kV and resolution of about 500 nm (ZEISS Company). Inductively coupled plasma optical emission spectroscopy (ICP-OES) was accomplished with a Varian Vista Pro CCD (Australia). UV–Vis spectra of the samples were obtained on a Hitachi UV-2910 spectrophotometer.

### Materials

All reagents and chemicals were used without further purification. Absolute ethanol (EtOH, 99.9%), concentrated ammonia (28 wt%), cetyltrimethylammonium bromide (CTAB, ≥ 98%), hydrochloric acid (HCl, 38%), tetraethyl orthosilicate (TEOS, 98%), 1,2-Bis(triethoxysilyl)ethane (BTEE, 97%), and Fumaric acid (HO_2_CCH = CHCO_2_H) used in this study were purchased from Sigma-Aldrich. Ferric chloride hexahydrate (FeCl_3_·6H_2_O) was bought from PubChem. Deionized water with a resistivity of 18.2 MΩ cm^−1^ was used in all experiments.

### Synthesis of double-shelled periodic mesoporous organosilica nanospheres

Double-shelled PMO nanospheres were obtained via a sol–gel process based on literature reports^140^. In a typical synthesis, 0.16 g of CTAB was combined with a mixed solution of ethanol (30 mL), concentrated ammonia (1.0 mL), and deionized water (75 mL) at 40 °C for half-hour. Afterward, a mixture containing BTSE (0.119 g, 0.33 mmol) and TEOS (0.116 g, 0.56 mmol) was quickly added to the above mixture under vigorous stirring (1100 rpm) at 40 °C and kept for 24 h. To obtain two-layered mesostructured organosilica spheres, a mixture of TEOS and BTSE with an initial molar ratio was added to the mixture of the previous step. After further stirring for 24 h at 40 °C, the slurry was collected by centrifugation and washed with ethanol. The periodic mesostructured organosilica spheres were re-dispersed in 360 mL of deionized water and then transferred to a Teflon-lined stainless-steel autoclave, which was heated in an airflow electric oven at 140 °C for 5 h. After cooling the autoclave to room temperature, the product was collected by centrifugation. Subsequently, by the solvent-extraction process containing a solution containing 180 mL of ethanol and 360 µL of concentrated HCl, CTAB templates were removed from the product. Finally, double-shelled ethane-bridged PMO nanospheres were obtained after washing with ethanol three times and drying under a high vacuum at 80 °C overnight.

### Synthesis of MIL-88A-Fe

To prepare the MIL-88A-Fe by the ultrasonic method, 1 mmol of FeCl_3_·6H_2_O (0.27 g) was dissolved in 10 mL mixture of the DMF and ethanolic solution previously prepared with a volume ratio of 4.5:1 while maintaining a constant molar ratio (NaOH: Fe = 0.8:1). In an equimolar ratio of FeCl_3_·6H_2_O, fumaric acid (1 mmol, 0.116 g) was dissolved in 5 mL of DMF. Afterward, both solutions were mixed and sonicated for a duration 10 min with a probe using 20 W and 10 kHz in continuous wave mode. Then, the as-synthesized MIL-88A-Fe rods were centrifuged and washed with DMF and ethanol several times and then dried under vacuum at 85 °C overnight^141^.

### Synthesis of double-shelled ethane-bridged PMO nanospheres/MIL-88A-Fe composite (DSS/MIL-88(A)-Fe) composite

**Scheme 1:**
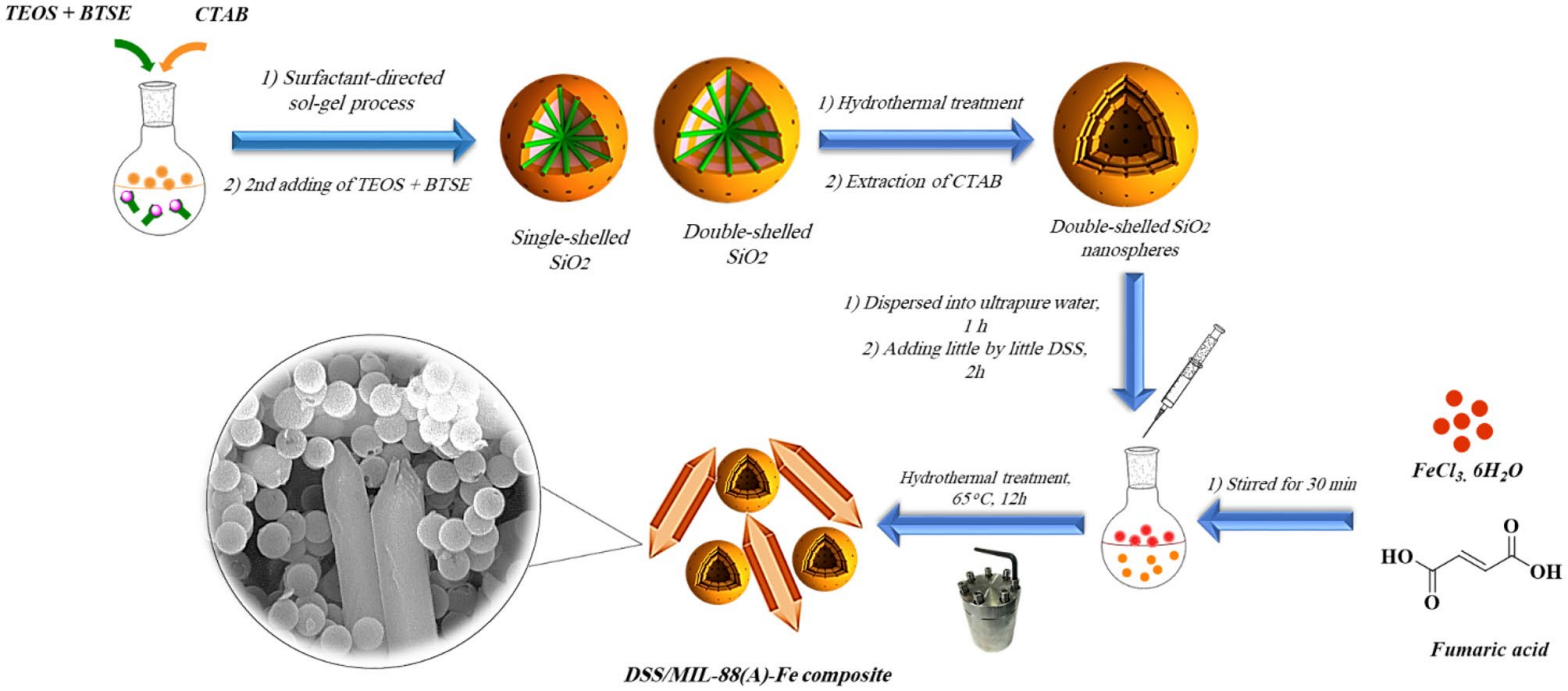
Preparation of the double-shelled periodic mesoporous organosilica nanospheres/MIL-88A-Fe composite.

As demonstrated in Scheme 1, the synthesis procedure of DSS/MIL-88(A)-Fe composite has proceeded through hydrothermal treatment. Firstly, a certain amount of the double-shelled ethane-bridged PMO powder (10 wt%) was dispersed into 20 mL ultrapure water in a bath sonicator at room temperature for 1 h. Afterward, 1 mmol of FeCl_3_·6H_2_O (0.27 g) and 1 mmol fumaric acid (0.116 g) were dissolved in a mixture of DMF and the ethanolic solution previously prepared with a proportion in volume 4.5:1 obtaining a 0.8:1 NaOH to Fe ratio in the reaction media. Then, double-shelled ethane-bridged PMO solution was gradually dropped into the above solution and sonicated for 15 min under the same conditions. Then the mixture was transferred into a 100 mL autoclave, sealed and heated to 65 °C for 12 h. The DSS/ MIL-88A-Fe composite was obtained by centrifugation and washed with ethanol several times and then dried under vacuum at 85 °C for 12 h.

### Adsorption experiments (preparation of Pb^2+^ ion solution)

#### Adsorption process

Various initial concentrations of Pb^2+^ solution (10, 20, 40, 60, 80, and 100 mg/L) were prepared by dissolving Pb(OAc)_2_ salt into deionized. Subsequently, DSS/MIL-88(A)-Fe composite (15 mg) was added in Pb^2+^ solution (50 mL) to adsorb Pb (II) at pH 6. The suspension was stirred during the test (5–150 min), and the temperature was controlled by a thermostat water bath (25 ± 2 °C). The adsorption kinetic data were obtained by sampling 5 mL of suspension at different periods during the experiment’s progress. The solid adsorbent composite was separated from the solution by filtration.

#### Analytical methods

The adsorption capacity (${q}_{e}$, mg g^−1^) of Pb^2+^ by the solid adsorbent composite at equilibrium and as well as the removal efficiency (%) (R) of Pb^2+^ was calculated by the following formula: where C_0_ (mg/L) is the initial concentration of Pb (II), and C_e_ (mg/L) is the equilibrium concentration in the liquid phase. V is the volume of solution (mL), and m is the amount of the adsorbent (mg).

1 $${q}_{e}=\frac{\left({C}_{0}-{C}_{e}\right)\times V}{m},$$

2 $$Removal \,efficiency\, \left(\%\right)= \frac{\left({C}_{0}-{C}_{e}\right)}{{C}_{0}}\times 100\%.$$

## Results and discussion

### Characterization of the DDS/MIL-88A-Fe composite

The preparation of the DSS/MIL-88(A)-Fe composite is demonstrated in Scheme 1. At the outset, monodispersed double-shelled periodic mesoporous silica nanospheres were obtained via sol–gel polymerization process^140^. SiO_2_ nanospheres two layers were achieved via a cetyltrimethylammonium bromide (CTAB) surfactant-directed sol–gel method by a mixture of silane precursors including tetraethyl orthosilicate (TEOS) and 1,2-bis(triethoxysilyl)ethane (BTSE) in water–ethanol solution. Double-shelled SiO_2_ nanospheres (DSS) are attained by adding a mixture of TEOS and BTSE into the reaction solution, with step-by-step addition at 24-h intervals. The successively grown DSS spheres were hydrothermally treated at 120 °C for 5 h for the transformation of the solid state to a hollow structure. SiO_2_ nanospheres two layers with the ordered radial mesochannels, could be obtained after the extract of CTAB surfactants from the shells by acidic ethanol. The preparation of DSS/MIL-88(A)-Fe composite adsorbent to remove Pb^2+^ from solution has proceeded through hydrothermal treatment. To a certain amount of FeCl_3_·6H_2_O in ultrapure water, fumaric acid was added under stirring. Then, the DSS nanospheres solution was gradually dropped into the above the solution and stirred forcefully for 2 h. Then the mixture was transferred into an autoclave to obtain the final nanocomposite.

After the successful synthesis of double-shelled periodic mesoporous organosilica nanospheres/MIL-88A-Fe composite, its structure was assessed using different spectroscopic methods, including Fourier Transform infrared spectroscopic analysis (FTIR), X-ray powder diffraction (XRD), Brunauer, Emmett and Teller (BET) surface area analysis, transmission electron microscopy (TEM), field emission scanning electron microscopy (FE-SEM), energy-dispersive X-ray spectroscopy, and inductively coupled plasma optical emission spectroscopy (ICP-OES).

**Figure 1:**
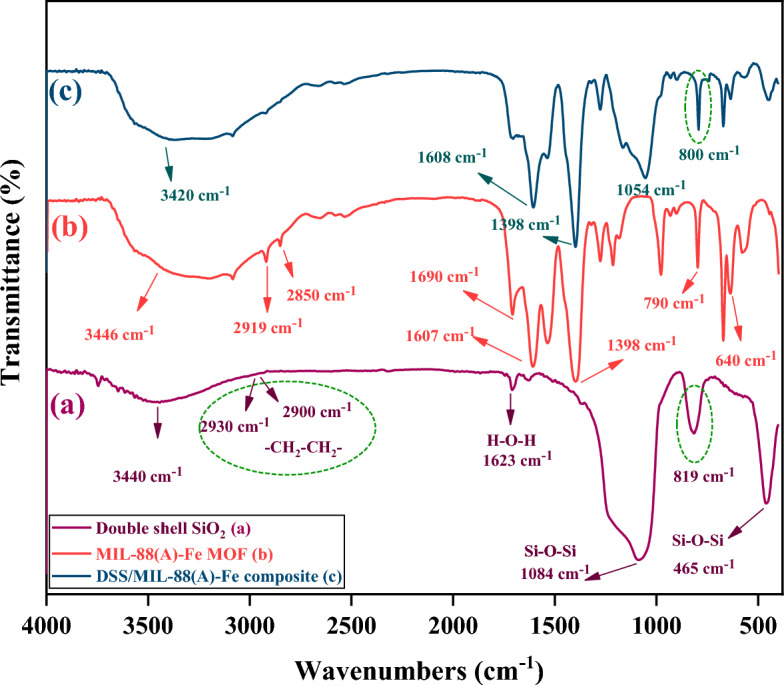
FTIR spectra of (a) DSS nanospheres, (b) MIL-88(A)-Fe MOF, and (c) DSS/MIL-88(A)-Fe composite.

The FTIR spectra of (a) DSS nanospheres, (b) MOF MIL-88(A)-Fe, and (c) DSS/MIL-88(A)-Fe composite, have been shown in Fig. 1. As it is evident from Fig. 1a, the typical absorption bands located at 1084, 819, and 465 cm^−1^ are assigned to the asymmetric, symmetric, and bending vibrations of the Si–O–Si bond, respectively. The FT-IR spectra of the DSS nanospheres showed absorbance bands of about 2930 cm^−1^, which can be assigned to the vibration of C–H bond in –CH_2_–CH_2_– group, clearly indicating the ethane-bridged frameworks.

The broad absorption band at 3440 cm^−1^ and the distinctive band at 1623 cm^−1^ can be related to the stretching and bending modes of the surface-attached hydroxyl groups (*ν* O–H) and adsorbed water molecules in DSS nanospheres, respectively^140^. In the FTIR spectrum of MOF MIL-88(A)-Fe (Fig. 1b), the band corresponding to the *ν*(C=C) for the fumarate ligand emerges as a sharp band in the region of 1690 cm^−1^^142^. Also, two influential bands at 1607, and 1398 cm^−1^ can be attributed to the asymmetric and symmetric vibration modes of the carboxyl group from fumaric acid, respectively. The characteristic peak at 790 cm^−1^ can be associated to the C–H bending vibration of the organic linker^143^. Besides, the absorption band at 640 cm^−1^ is allocated to carbonyl group^144,145^. In the case of DSS/MIL-88(A)-Fe composite, as can be observed in Fig. 1c, the characteristic stretching vibration of O–H (attributed to the double-shelled SiO_2_) is found at 3420 cm^−1^, which is covered by O–H vibrational mode of water content (related to the adsorption of moisture in the air to MOF. Furthermore, the definite structure of the nanocomposite was corroborated by the advent of the absorption bands at 1608 and 1398 cm^−1^ (attributed to coordination between the carboxyl group and Fe^3+^), and absorption bands at 1054 and 800 cm^−1^ (associated with the vibrations of the Si–O–Si bond). The obtained results approved that the spectral information agrees with the XRD analysis results described in the following.

**Figure 2:**
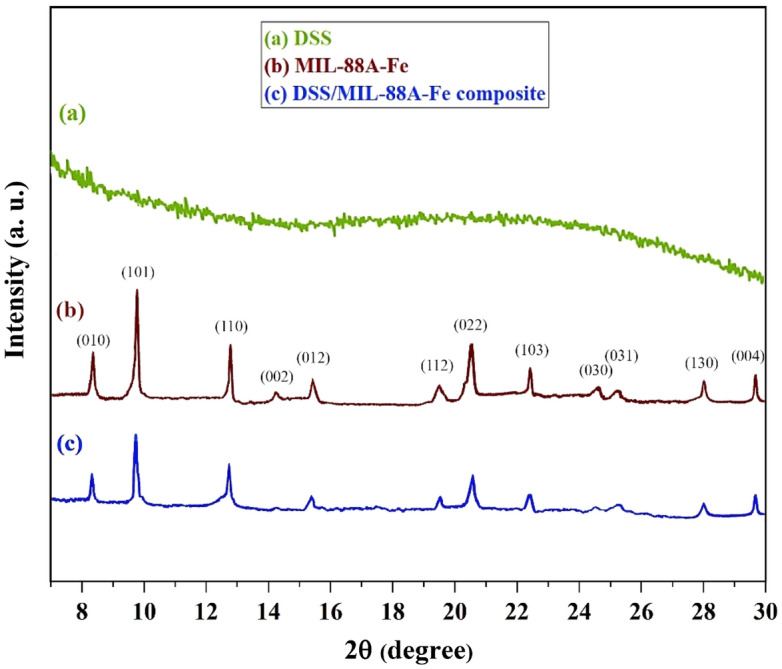
XRD patterns of DSS nanospheres (a), MIL-88(A)-Fe MOF (b), and DSS/MIL-88(A)-Fe composite (c).

In order to confirm the crystalline structure of (a) DSS nanospheres, (b) MIL-88(A)-Fe MOF, and (c) DSS/MIL-88(A)-Fe composite, the powder X-ray diffraction (PXRD) technique was performed, and the results are shown in Fig. 2. It can be observed (Fig. 2a), that the amorphous structure of double-shelled SiO_2_ (DSS) nanospheres (I) exhibits a broad diffraction peak at 2θ = 22.5°^146^. For the MIL-88(A)-Fe MOF, the major distinct peaks are located at 2θ = 8.4°, 9.9°, 12.8°, 15.3°,15.9°, 21.1°, and 22.5° related to the (010), (101), (110), (002), (012), (022) and (103) crystal planes (Fig. 2b)^141,147^. In the XRD pattern of the DSS/MIL-88(A)-Fe composite, all peaks were well-matched with those of their corresponding pure MIL-88(A)-Fe MOF. Notably, the diffraction peak at 2θ = 8.5° attributed to the (100) crystallographic facet developed more than that of pure MIL-88(A)-Fe (Fig. 2c). The shift might be due to the double-shelled periodic mesoporous organosilica nanospheres that controlled the MIL-88(A)-Fe MOF crystal orientation in the modified composite. The XRD results suggested that the pure MIL-88(A)-Fe MOF and desired composite were successfully synthesized.

**Figure 3:**
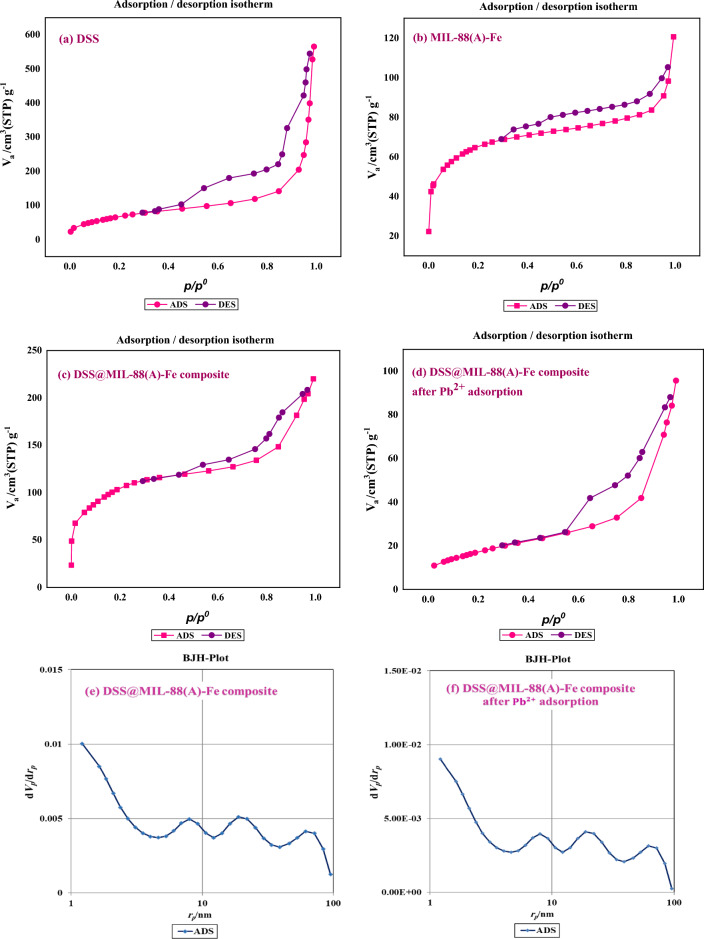
N_2_ adsorption–desorption analysis of DSS nanospheres (**a**), MIL-88(A)-Fe MOF (**b**), DSS/MIL-88(A)-Fe nanocomposite (**c**), and DSS/MIL-88(A)-Fe nanocomposite after Pb^2+^ adsorption (**d**). BJH plots of DSS/MIL-88(A)-Fe nanocomposite (**e**), and DSS/MIL-88(A)-Fe nanocomposite after Pb^2+^ adsorption (**f**).

It is known that the BET surface area and pore structure of the as-synthesized nanocomposite are substantial factors, which influence the catalytic activity. To gain further insights into the textural properties (the pore-size distributions and BET surface areas), N_2_ adsorption-desorption analysis of DSS nanospheres (a), MIL-88(A)-Fe MOF (b), DSS/MIL-88(A)-Fe nanocomposite (c), and DSS/MIL-88(A)-Fe nanocomposite after Pb^2+^ adsorption (d) were presented in Fig. 3. As seen in Fig. 3a, DSS nanospheres exhibit typical type IV isotherm with a large hysteresis loop indicating the presence of mesoporous structure with a high surface area of about 244.87 m^2^ g^−1^ and pore volume of 0.864 cm^3^ g^−1^. It was found in Fig. 3b that the hysteresis loops in the isotherm curve of MIL-88(A)-Fe MOF can be attributed to typical type IV isotherm with H3 hysteresis loop^148^. Furthermore, the derived BET (obtained from Brunauer–Emmett–Teller (BET) theory) surface area and pore volume were estimated to be 236 m^2^ g^−1^ and 0.180 cm^3^ g^−1^, respectively. As it is evident from the data summarized in Table 1, the BET surface area of DSS/MIL-88(A)-Fe nanocomposite is larger than the pure DSS and MOF (Fig. 3c). The larger pore volume of DSS/MIL-88(A)-Fe nanocomposite compared to the pure DSS and MOF offered a mesoporous architecture for composite samples, providing a suitable pathway for mass transport (Table 1). Moreover, the mean pore volume of DSS/MIL-88(A)-Fe nanocomposite (2.919 nm) is slightly lower than those of the pure DSS (14.115 nm) and MOF (3.056 nm), which is probably due to the synergistic effect and implies the successful combination of MOF and PMOs. Furthermore, investigating on the Barrett–Joyner–Halenda (BJH) pore size distribution which was calculated using the adsorption branch (presented in Fig. 3e) clearly shows three peaks centered at 8, 19 and 61 nm, corresponding to the mesoporous of the shell, hollow void (the shell-in-shell distance) and also macro porous of MOF, respectively. By comparing the BJH before and after Pb^2+^ adsorption, the conclusion could be derived that no significant changes were observed in the pore size distribution (Fig. 3e,f).

**Table 1:** Specific surface area (S_BET_), total pore volume (*V*_*t*_), and pore diameter of DSS nanospheres (a), MIL-88(A)-Fe MOF (b), DSS/MIL-88(A)-Fe nanocomposite (c) and DSS/MIL-88(A)-Fe nanocomposite after Pb^2+^ adsorption (d).

Samples	S_BET_ (m^2^ g^−1^)	Total pore volume (V_t_)	Mean pore diameter (nm)
DSS nanospheres (a)	244.87	0.864	14.115
MIL-88(A)-Fe MOF (b)	236	0.180	3.056
DSS/MIL-88(A)-Fe nanocomposite (c)	312.87	0.228	2.919
DSS/MIL-88(A)-Fe nanocomposite after Pb^2+^ adsorption (d)	62.655	0.147	0.943

**Figure 4:**
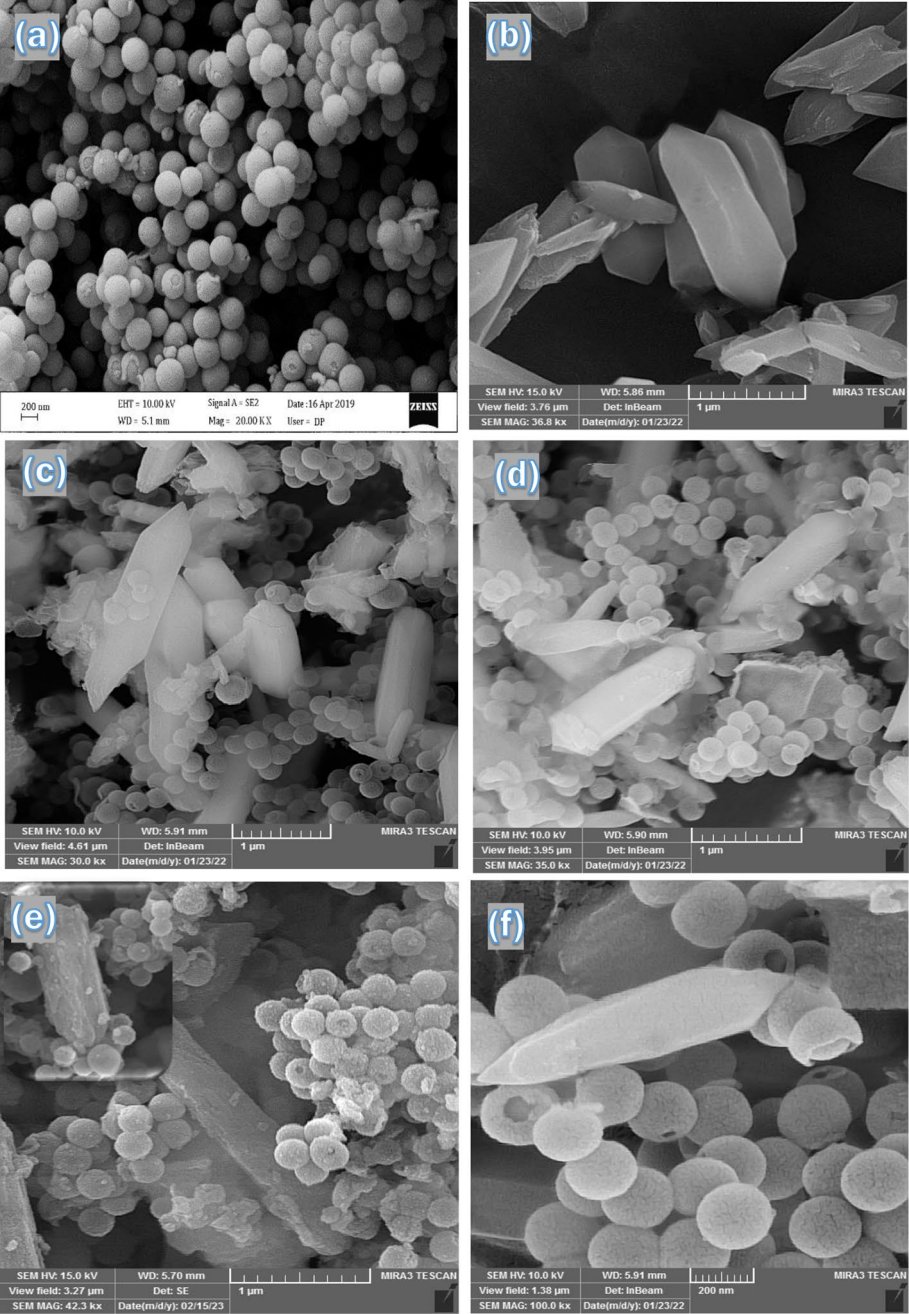
SEM images of DSS nanospheres (**a**), MIL-88(A)-Fe MOF (**b**), DSS/MIL-88(A)-Fe nanocomposite (**c,d**), after Pb^2+^ adsorption (**e**), and reusability of DSS/MIL-88(A)-Fe nanocomposite after 4 adsorption–desorption cycles (**f**).

The morphological studies of DSS/MIL-88(A)-Fe nanocomposite was performed by investigating the field emission scanning electron microscopy (FE-SEM) and transmission electron microscopy (TEM). The obtained results are presented in Fig. 4. In order to testify the well synthesized DSS/MIL-88(A)-Fe nanocomposite, initially we examined the pure DDS nanospheres and MIL-88(A)-Fe MOF by FE-SEM analysis. As can be observed from Fig. 4a, DDS exhibits a uniformly spherical shape, monodisperse size distribution, and high surface area with particle sizes of about 280 nm. Also, the original MIL-88(A)-Fe MOF shows well-crystallized rods with a hexagonal face and an average size of 1.2 μm (Fig. 4b). The formation of DSS/MIL-88(A)-Fe nanocomposite is obviously confirmed from the FE-SEM images (Fig. 4c,d). As revealed by Fig. 4c,d, the obtained DSS/MIL-88(A)-Fe nanocomposite preserved the original shape from the parent DSS and MOF with an average size of 280 nm and 1.1 μm, respectively. Interestingly, the nanocomposite structure shows the formation of a regular arrangement of DDS nanospheres, as indicated in Fig. 4a, along with the formation of a hexagonal-rods structure with dimensions in the range of the former MOF morphologies. Moreover, to investigate the effect of Pb^2+^ adsorption on the morphology, the FE-SEM image of DSS/MIL-88(A)-Fe nanocomposite after adsorption was recorded and illustrated in Fig. 4e. By comparing the FE-SEM images of DSS/MIL-88(A)-Fe nanocomposite before (Fig. 4c,d) and after (Fig. 4e) adsorption of Pb^2+^, it could be concluded that Pb^2+^ ions adsorbed into the surface of DSS/MIL-88(A)-Fe nanocomposite.

**Figure 5:**
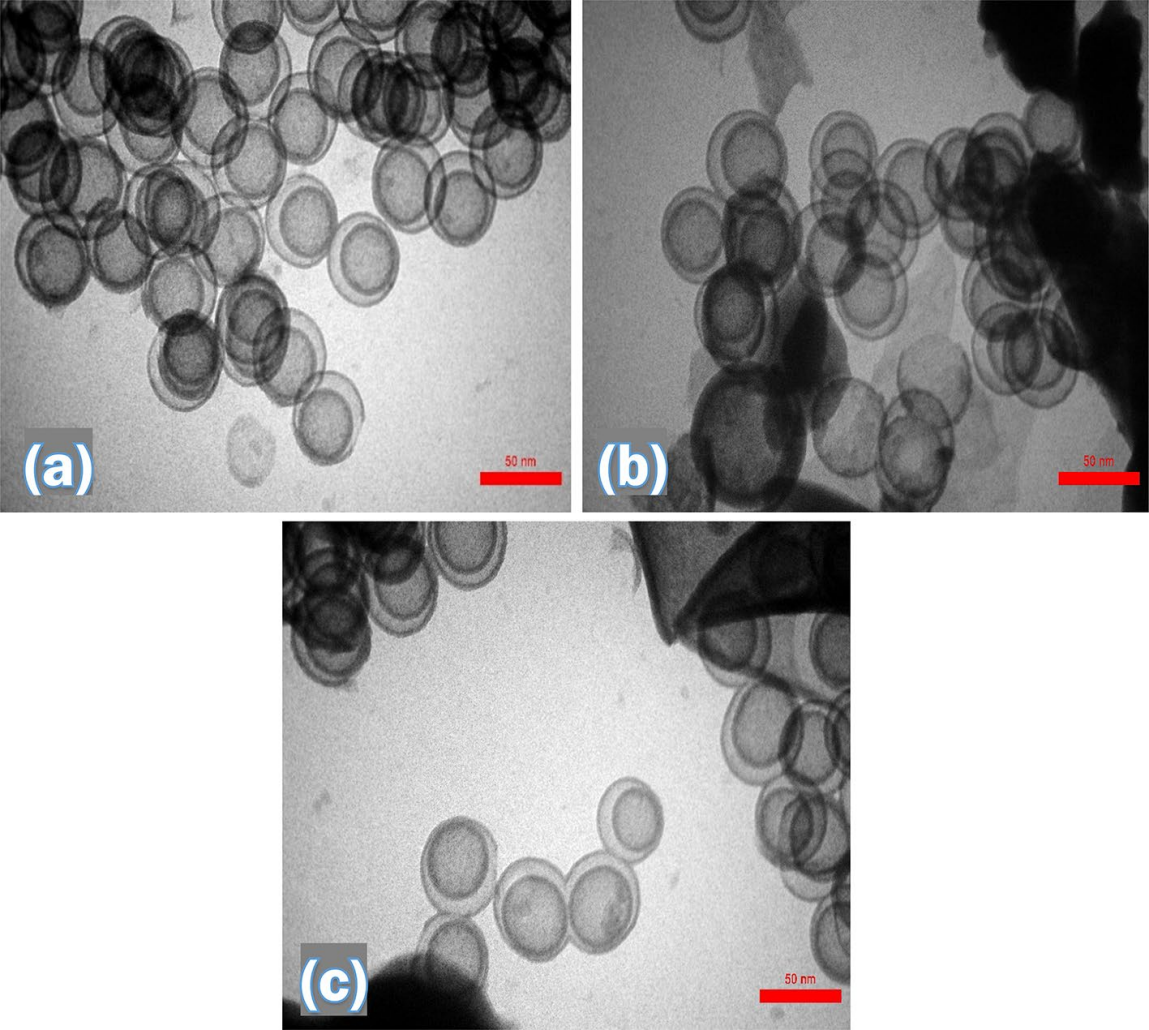
TEM images of DSS (**a**), and DSS/MIL-88(A)-Fe nanocomposite (**b,c**).

For further insight on the morphology of the synthesized DSS/MIL-88(A)-Fe nanocomposite, the TEM images were surveyed and presented in Fig. 5a–c. The TEM images of the nanocomposite confirm the existence of bright and dark areas in the double-shelled structures. Furthermore, as it is evident from Fig. 5a,b, the cavities observed from TEM images suggested the presence of mesoporous channels on the surface of the DSS nanospheres. Of note that the formation of the hexagonal-rods structure of MIL-88(A)-Fe MOF was clearly observed in Fig. 5c. Also, the thickness of the external layer, internal layer and the hollow void (the shell-to-shell distance) was estimated to be ∼13, ∼ 20 and ∼33 nm, respectively. Accordingly, the overall outer diameter is about ∼280 nm for DSS nanospheres which is very close to the FE-SEM data.

**Figure 6:**
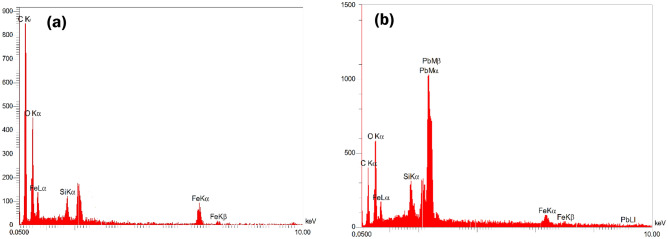
EDX of DSS/MIL-88(A)-Fe nanocomposite (**a**), and after adsorption of Pb^2+^ (**b**).

Surveying the EDX spectrum confirmed the existence of C, O, and Fe elements in the nanocomposite structure (Fig. 6a). After adsorption, the Pb^2+^ signals have been detected in the used adsorbent surface (Fig. 6b).

**Figure 7:**
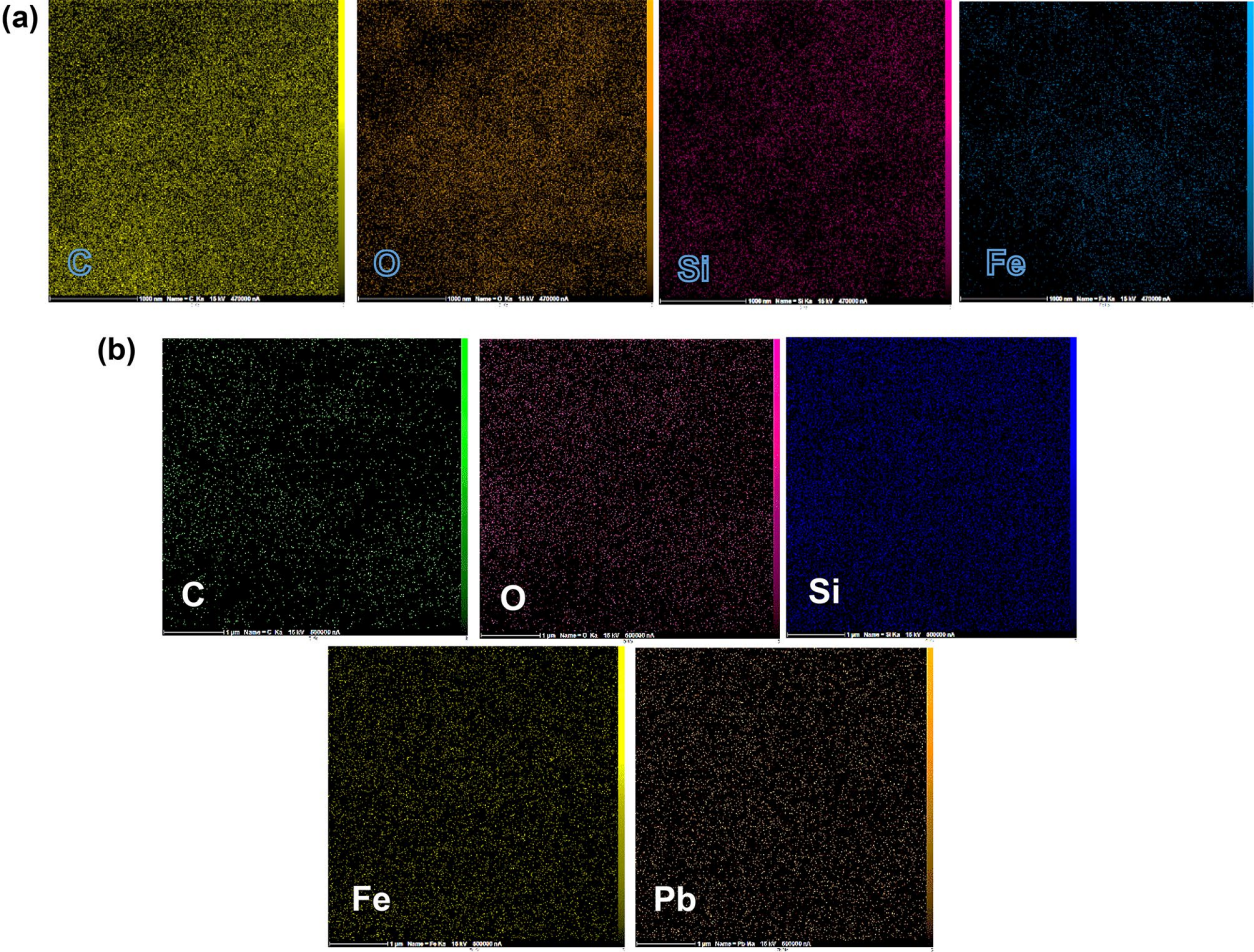
EDX-mapping of DSS/MIL-88(A)-Fe nanocomposite (**a**), and after adsorption of Pb^2+^ (**b**).

To further evaluate the DSS/MIL-88(A)-Fe nanocomposite, EDX-mapping analysis was performed and the presence of C, O, Si, and Fe with uniform distributions was approved (Fig. 7a). In addition, after Pb^2+^ adsorption, the elemental distributions were almost identical. These findings evidenced that Pb^2+^ had successfully attached to the adsorbent surface (Fig. 7b).

### Effect of time and initial contact concentration of Pb^2+^

**Figure 8:**
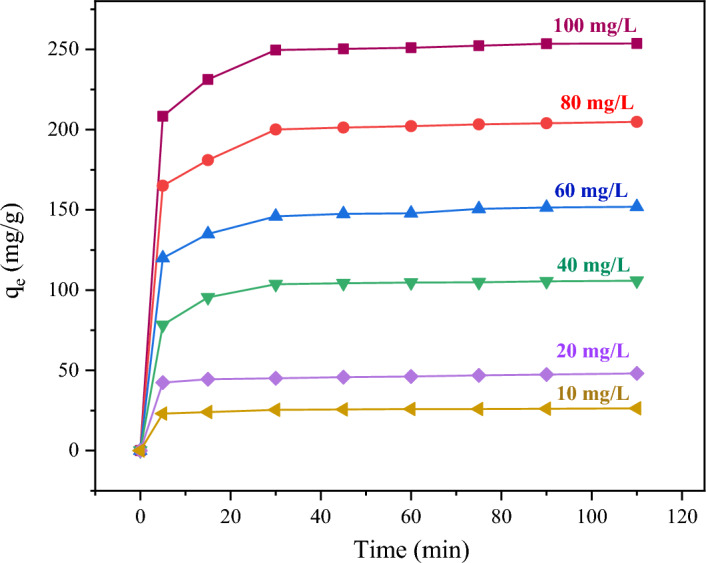
Effect of contact initial concentration of Pb^2+^ and time on its adsorption.

In this study, the removal of Pb^2+^ from aqueous solution has been investigated using double-shelled periodic mesoporous organosilica nanospheres/MIL-88A-Fe composite. In order to check the adsorption of lead ions from an aqueous solution, in the first, to the 50 mL solutions of Pb^2+^ with varying concentrations (10, 20, 40, 60, 80, 100 mg/L), 15 mg DSS/MIL-88A-Fe composite was separately added at pH 6. Then, initial concentrations of Pb^2+^ were measured at an interval of 5, 15, 30, 45, 60, 75, 90, and 110 min at room temperature. The effects of contact time and initial concentration of Pb^2+^ on the adsorption of Pb^2+^ by DSS/MIL-88A-Fe were studied. It was found from Fig. 8 that the adsorption capacity of Pb^2+^ with an initial mass concentration of 10–100 mg/L on DSS/MIL-88A-Fe composite reached an adsorption equilibrium within 30 min.

**Figure 9:**
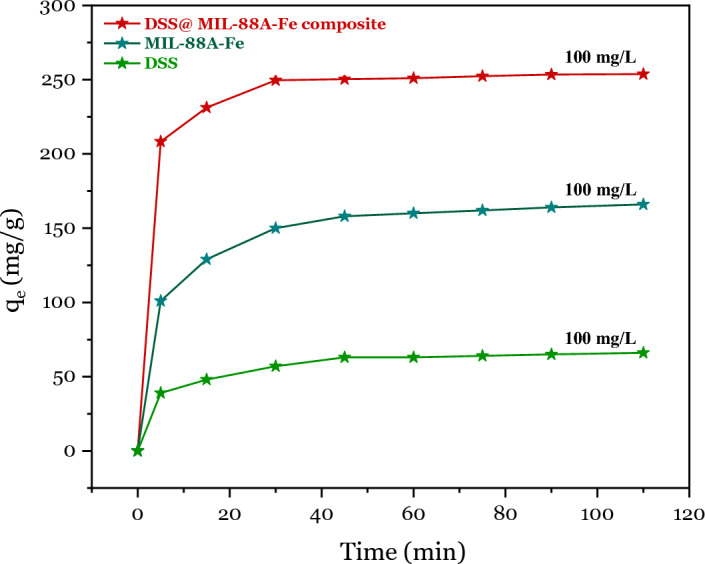
Pb^2+^ adsorption isotherms for DSS, MIL-88A-Fe and DSS/MIL-88A-Fe composite at room temperature with a concentration of 100 mg/L of Pb^2+^ solution.

From Fig. 9, DSS/MIL-88A-Fe composite has the highest adsorption capacity compared with MIL-88A-Fe and DDS, which is attributed to the higher surface area and more numerous active sites for adsorption of Pb^2+^ ions (Pb^2+^ solution with a concentration of 100 mg/L). It became apparent that the adsorption capacity increased with an increase in the initial Pb^2+^ concentration. This is due to the increase in the driving force of the concentration gradient with the increase in the initial Pb^2+^ concentration.

### Effect of adsorbent dosage

**Figure 10:**
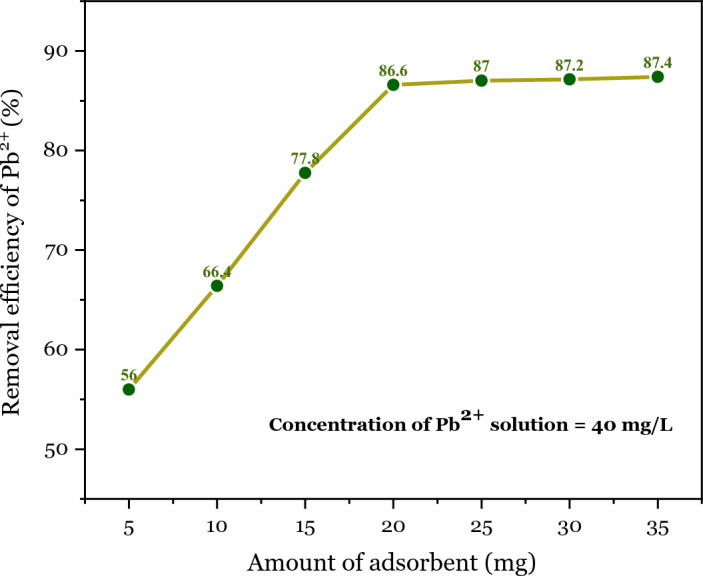
Effect of adsorbent dosage on adsorption capacity and removal efficiency of Pb^2+^.

The effect of adsorbent dosage on the removal efficiency of Pb^2+^ by DSS/ MIL-88A-Fe composite was investigated. For this purpose, 50 mL of Pb^2+^ solution with a concentration of 40 mg/L, and 5–35 mg of DSS/MIL-88A-Fe composite were added at room temperature and stirred for 30 min. As can be seen from Fig. 10, with the increase of adsorbent dosage, the percentage removal initially increases, which could be due to the increase of the total surface area of the adsorbent, and the adsorption site number of the adsorbent in a certain amount of solution. But beyond a value of 20 mg, the percentage removal reaches an almost constant value. This may be due to an overlapping of adsorption sites and, consequently, of the adsorbent particles overcrowding. Maximum removal of 86.6% was observed at an adsorbent dosage of 20 mg/L (for economic purposes) at pH 6**.**

### Effect of pH value

**Figure 11:**
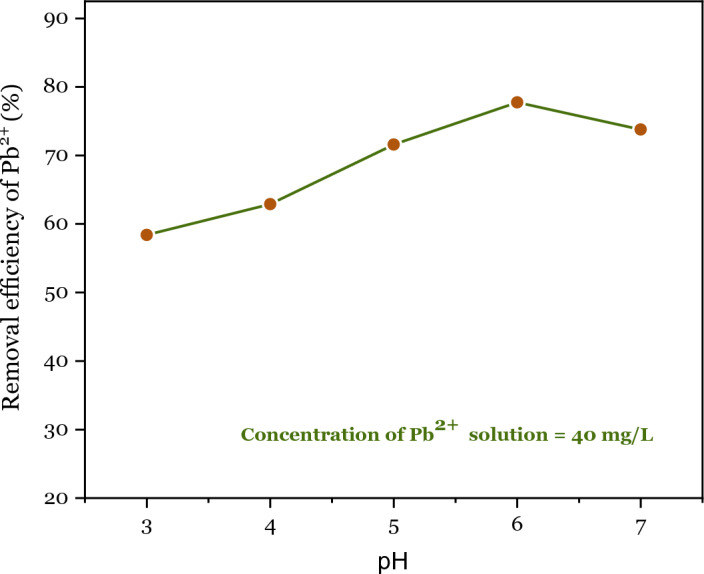
Effect of pH value on adsorption capacity of Pb^2+^.

It is well-known that the pH value of the solution is an essential factor affecting the adsorption performance of Pb^2+^. For this purpose, different pH values (3, 4, 5, 6, and 7) affecting the adsorption performance of Pb^2+^ by DSS/MIL-88A-Fe were investigated (Fig. 11). The adsorption capacity of Pb^2+^ on composite depends on the pH value, which increased slowly with an increase of pH until pH 6.

The surface of the DSS/MIL-88A-Fe composite is protonated in the acidic solutions (pH 3, 4, and 5), which contains a positive charge causing electrostatic repulsion between DSS/MIL-88A-Fe and Pb^2+^. This phenomenon demonstrated that a large amount of H^+^ ions competed with Pb^2+^ ions for the adsorption sites in the composite in an acidic environment^149^. Thus, the adsorption capacity of Pb^2+^ by the adsorbent DSS/MIL-88A-Fe composite was reduced. When the pH exceeds 6, Pb^2+^ ions quickly form Pb^2+^ hydroxide (precipitation) with OH^−^^150^. Hence the optimum pH of 6 was selected for the subsequent adsorption experiments.

### Adsorption isotherm

To obtain mechanism information on the adsorption process, adsorption isotherm models are important for designing the adsorption system. The adsorption capacity of Pb^2+^ by DSS/MIL-88A-Fe composite was evaluated using Langmuir (3), Freundlich (4), and Temkin (5) isothermal adsorption model to find the interaction between DSS/MIL-88A-Fe composite and Pb^2+^. The corresponding equations of isothermal adsorption models are as follows;

3 $$\frac{{C}_{e}}{{q}_{e}}= \frac{1}{{{k}_{L}q}_{L }}+ \frac{{C}_{e}}{{q}_{L}},$$

4 $$\mathrm{ln}\,{q}_{e}= \mathrm{ln}\,{k}_{f}+ \frac{1}{n}\mathrm{ln}\,{C}_{e},$$

5 $${q}_{e}= \frac{RT}{{b}_{T}}\mathrm{ln}\,{k}_{T}+ \frac{RT}{{b}_{T}}\mathrm{ln}\,{C}_{e},$$

where ${q}_{e}$(mg/g) is the equilibrium adsorption capacity of Pb^2+^ in solution, ${q}_{L}$(mg g^−1^) represents the maximum adsorption capacity, K_L_ (L mg^−1^) is the Langmuir constant ascribed to the affinity of the binding sites between the adsorbent and the target substance. K_F_ ((mg g^−1^)/(mg g^−1^)^1/n^) and n ((mg^(1−(1/n))^ L^(1/n)^ g^−1^) are Freundlich constants representing adsorption capacity and adsorption intensity, respectively. R is the gas constant (8.314 J/mol⋅K), T is the temperature (K).

**Figure 12:**
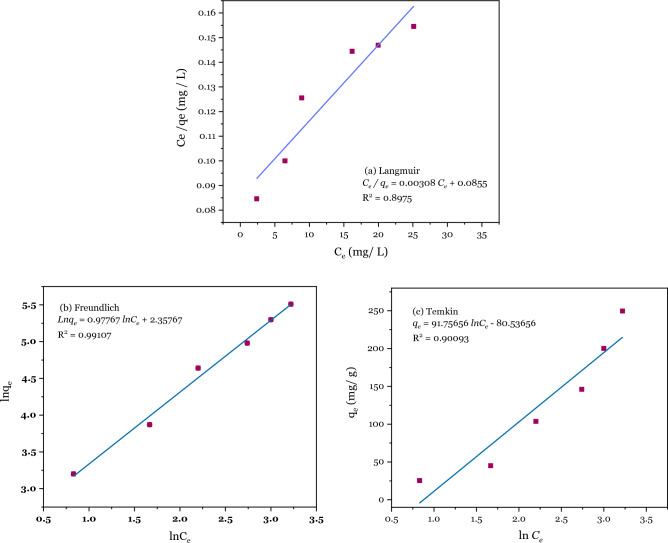
Linear plot for (**a**) Langmuir, (**b**) Freundlich, and (**c**) Temkin adsorption isotherm models.

Three models of experimental data from adsorption processes were applied to explain the Pb^2+^ adsorption mechanism between the liquid and adsorbent phases. The fitting results of the adsorption of Pb^2+^ are shown in Fig. 12. The parameters of the models are listed in Table 2.

**Table 2:** Langmuir (a), Freundlich (b), and Temkin adsorption isotherm parameters of Pb^2+^ adsorption on DSS/MIL-88A-Fe composite.

Langmuir isotherm			Freundlich isotherm			Temkin isotherm		
${q}_{L}$	${K}_{L}$	R^2^	$\frac{1}{n}$	${k}_{f}$	R^2^	${b}_{T}$	${k}_{T}$	R^2^
342.67	0.0341	0.8975	0.9770	10.5663	0.99107	27.0015	0.4160	0.9009

The Langmuir model is normally associated with monolayer adsorption characteristics and the energy level of a homogeneous system, which has no following interaction between adsorbed species^151^. The Freundlich model is generally an empirical one related to heterogeneous systems and applied to multi-layer adsorption of the adsorbent^152^.

The Temkin model is commonly described by a uniform distribution of binding energies to explain adsorbate–adsorbent interactions on adsorption sites^153^.

The most favourable isothermal adsorption model was provided by the Freundlich model, as it yielded a higher R^2^ value in contrast to the value of the correlation coefficient between the Langmuir and Temkin models. The adsorption behaviour of Pb^2+^ by DSS/MIL-88A-Fe composite mainly occurred on heterogeneous pores or surfaces as the main adsorption sites, and multi-layer adsorption could exist.

$\frac{1}{\mathrm{n}}$ is a constant reflecting the Pb^2+^ adsorption intensity by the adsorbent; if the value of $\frac{1}{\mathrm{n}}$ sits between 0.1 and 1.0, the process of adsorption is favourable^154^.

### Effect of temperature and thermodynamic parameters

Temperature is one of the essential factors that influence the adsorption capacity of composite on absorbing Pb^2+^. The effect of temperature on the adsorption of Pb^2+^on DSS/MIL-88A-Fe composite was investigated at three temperatures at 298, 308, and 318 K. Obviously, on increasing the temperature, the adsorption capacity of the adsorbent on absorbing Pb^2+^ increased. This showed that the adsorption process was endothermic.

To further investigate the thermodynamic features, the thermodynamic parameters such as Gibb’s free energy change (ΔG°, kJ/mol), enthalpy change (ΔH°, kJ/mol), and entropy change (ΔS°, J/(mol·K)) were calculated using the following equations:

6 $$\Delta \mathrm{G}^\circ =-\mathrm{RT ln}{\mathrm{K}}_{\mathrm{c}}= -\mathrm{RTln}\left(\frac{{\mathrm{q}}_{\mathrm{e}}}{{\mathrm{C}}_{\mathrm{e}}}\right),$$

7 $$\Delta \mathrm{G}^\circ = \Delta \mathrm{H}^\circ -\mathrm{T}\Delta \mathrm{S}^\circ ,$$

8 $$\mathrm{ln}\begin{array}{c}{\mathrm{K}}_{\mathrm{c}}= \frac{\Delta \mathrm{H}^\circ }{\mathrm{RT}}+\\ \end{array}\frac{\Delta \mathrm{S}^\circ }{\mathrm{R}},$$

where R is the universal gas constant (8.314 J/mol K); T is the Kelvin temperature (K); K_c_ represents the thermodynamic equilibrium constant.

**Figure 13:**
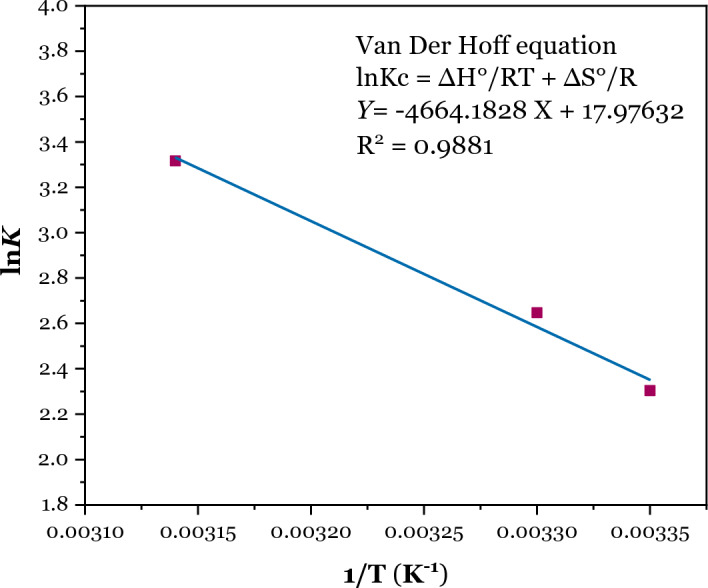
Van Der Hoff equation. Experimental conditions: [Pb^2+^] = 80 mg L^−1^, pH 6.0, adsorbent dose = 15 mg, solution volume = 50 mL; time = 30 min.

The ΔH° and ΔS° values were acquired from the slopes, and intercepts of the Van’t Hoff curve. Results are shown in Fig. 13 and the thermodynamic parameters are provided in Table 3.

**Table 3:** Adsorption thermodynamic parameters.

Temperature (K)	ΔG^0^ (kJ mol^−1^)	ΔH^0^ (kJ mol^−1^)	ΔS^0^ (J mol^−1^ K^−1^)
298	− 5.76	38.77	149.455
303	− 6.51		
318	− 8.75		

The negative values of ΔG° indicated the adsorption of Pb^2+^ on DSS/MIL-88A-Fe composite was spontaneous. Moreover, with the elevated temperature, the absolute value of ΔG° increased, revealing that high temperature can promote the adsorption process.

The positive values of ΔH° suggest that the adsorption process was endothermic, in nature, whereas the positive value of entropy change (ΔS°) reveals the increase in randomness at the solid/solution interface during the adsorption of Pb^2+^. Therefore, the adsorption was an endothermic, and spontaneous process.

### Adsorption kinetics

The adsorption kinetics of Pb^2+^ on DSS/MIL-88A-Fe composite using 80 mg L^−1^ Pb^2+^ solution of pH 6 at room temperature were also studied. As shown in Fig. 8, the fast Pb^2+^ adsorption process on DSS/MIL-88A-Fe composite in the first 5 min may be due to the presence of sufficient active adsorption sites available on the surface of the adsorbent. The adsorption was almost attained equilibrium within 30 min.

To study the adsorption kinetics and accurately interpret the adsorption behavior of Pb^2+^ adsorption on DSS/MIL-88A-Fe composite, four types of kinetic models, including the pseudo-first-order (9), pseudo-second-order (10), Elovich model (11), and particle diffusion (12) are expressed as follows^155,156^;

9 $$\mathrm{ln}\,\left({q}_{e}-{q}_{t} \right)=\mathrm{ln}\,{q}_{e}- {K}_{1}. t,$$

10 $$\frac{t}{{q}_{t}}= \frac{1}{{{k}_{2}. {q}_{e}^{2}}}+ \frac{t}{{q}_{e}},$$

11 $${q}_{t}= \frac{1}{\beta }\mathrm{ln}\,(\alpha .\beta )+\frac{1}{\beta }\mathrm{ln}t,$$

12 $${q}_{t}= {K}_{i} . {t}^{0.5}+ a,$$

where ${q}_{e}$ and ${q}_{t}$ are the adsorption capacity at equilibrium and time (mg g^−1^), *K*_1_ (min^−1^), and *K*_2_ [g (mg min)^−1^] are adsorption rate constant of pseudo-first-order and pseudo-second-order kinetics, respectively. *K*_*i*_ (mg/g ${\mathrm{min}}^{0.5})$ and *a* are adsorption rate constant of intra-particle diffusion, and intra-particle diffusion constants that reflecting boundary layer effect, respectively. $\alpha$[mg (g min)^−1^] and $\beta$(g mg^−1^) illustrate the initial constant adsorption and desorption constants, respectively^157–159^.

The adsorption process usually has various steps and out of which the slowest step controls the rate of the adsorption process.

**Figure 14:**
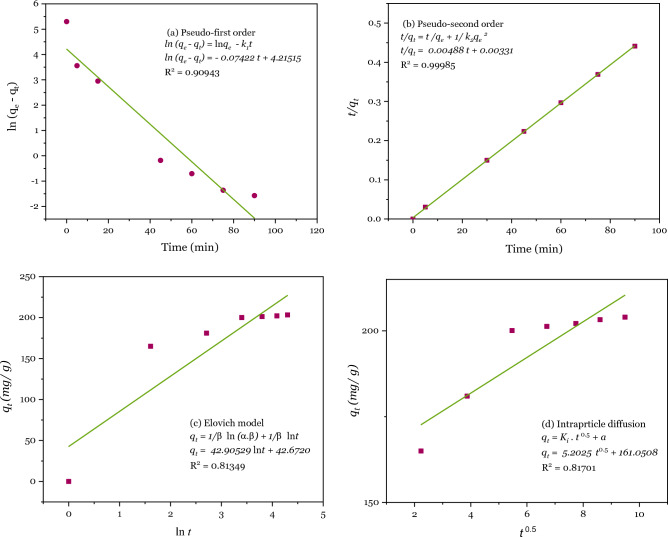
Plots of pseudo-first-order kinetic model for the adsorption of Pb^2+^ (**a**), pseudo-second-order kinetic model for the adsorption of Pb^2+^ (**b**), Elovich kinetic model for the adsorption of Pb^2+^ (**c**), and intra-particle-diffusion kinetic model for the adsorption of Pb^2+^ (**d**). Conditions: T = 398 K, adsorbent = 15 mg/L, metal = 80 mg/ L, pH 6, and contact time = 90 min.

The experimental data were fitted by using the above four adsorption kinetic models. According to the fitting results in Fig. 14a–d, and Table 4, it is evident that the R^2^ value of the pseudo-second-order dynamics model is greater than that of the other three models (the correlation coefficient R^2^ of the pseudo-second-order dynamic model is equal 0.998). This result indicates that the adsorption rate on the surface of the adsorbent is the rate-determining step and the adsorbent surface corresponds to a heterogeneous system.

**Table 4:** The parameters of four types of kinetic models.

Kinetic model	Parameter	Value
(a) Pseudo-first-order model	q_e_ (mg/g)	67.704
	K_1_ (min^−1^)	− 0.07422
	R^2^	0.90943
(b) Pseudo-second-order model	q_e_ (mg/g)	204.9180
	K_2_ [g (mg min)^−1^]	0.007
	R^2^	0.9985
(c) Elovich model	$\alpha$[mg (g min)^−1^]	11.55
	$\beta$(g mg^−1^)	0.0233
	R^2^	0.9302
(d) Intraparticle diffusion model	K_i_ [mg (g min^0.5^)^−1^]	5.2025
	R^2^	0.81701

### Pb^2+^adsorption mechanism

According to the above analysis and characterization, a possible mechanism for Pb^2+^ removal is suggested. To further understand the Pb^2+^ adsorption process and the composite-heavy metal interaction, the zeta potential of the DSS and MIL-88(A)-Fe was measured at pH 6. On the basis of the obtained results, the surface charge of DDS was negative (− 40 mV) indicating the electrostatic attraction enhancing for more Pb^2+^ adsorption. So, the mechanism of adsorption between DSS-heavy metals is due to the electrostatic attraction of unlike charges at pH 6.

On the other hand, DSS has provided a high specific surface area and mesoporous channel microstructure in which many hydroxyl-functional groups were exposed on the surface of DSS materials as active sites for the adsorption of heavy metal ions. The adsorption has occurred mainly through electrostatic interactions between the surface hydroxyl group of DSS and the heavy metal ions.

Furthermore, the Pb^2+^ adsorption mechanism in the solution via MIL-88(A)-Fe could readily happen by ion exchange protons on the surface of the adsorbent with Pb^2+^ (This observation has been confirmed by comparison before and after Pb^2+^ adsorption through FTIR and SEM analysis).

It could be understood that the Pb^2+^ adsorption mechanism involved competitive ion exchange with MIL-88(A)-Fe and electrostatic interactions with the DSS of the composite. Also, the Pb^2+^ adsorption mechanism could be occurred from binding to open metal sites on the MOF of composite (a pore-filling mechanism) or interacting with active sites on the surface of DSS containing hydroxyl-functional groups.

The MIL-88(A)-Fe showed a positive surface charge (+ 30 mV). It is thus concluded, from this standpoint, the interaction between the DDS nanoparticles and MIL-88(A)-Fe is through electrostatic attraction to form a composite material denoted as DSS/MIL-88(A)-Fe.

### Study of DSS/MIL-88(A)-Fe composite recycling

**Figure 15:**
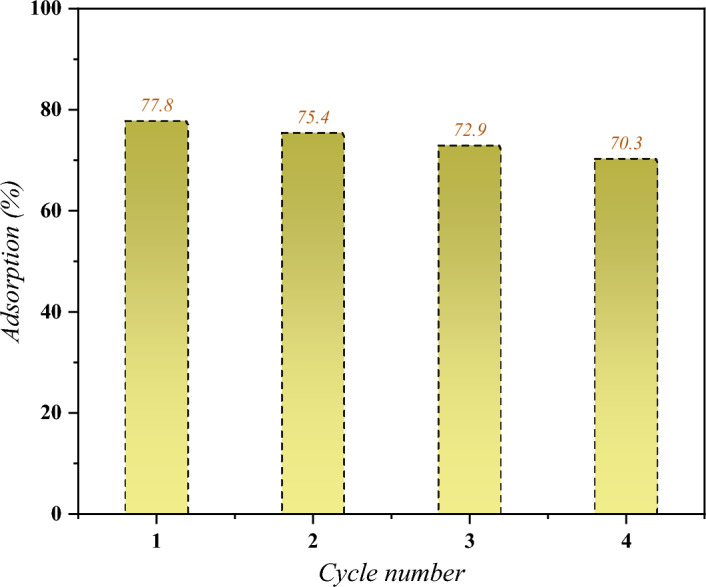
Cycle performance of DSS/MIL-88(A)-Fe composite for Pb^2+^ adsorption.

The recyclability of the adsorbent is an important index, both economically and environmentally. In order to evaluate the recycling ability of DSS/MIL-88(A)-Fe composite, the adsorbent was placed in an HCl solution with a concentration of 0.05 M and stirred for 2 h, followed by filtering and washing with nitric acid for 3 times. Afterward, the composite was cleaned with deionized water for several times and activated at 85 °C for 10 h to adsorb Pb^2+^ again in 40 mg L^−1^ solution. The effect of DSS/MIL-88(A)-Fe composite cycling times on adsorption capacity is revealed in Fig. 15. The adsorption capacity of Pb^2+^ by DSS/MIL-88(A)-Fe composite decreases slightly after 4 periods of regeneration to reuse the adsorbent (from 77.8% at the first cycle to 70.3% at the fourth cycle). The decrease in adsorption capacity during repeated use could be caused by the mass loss of the composite adsorbent in acid treatments. The results demonstrate good recycling capability in the cyclic adsorption process for Pb^2+^ adsorption.

**Figure 16:**
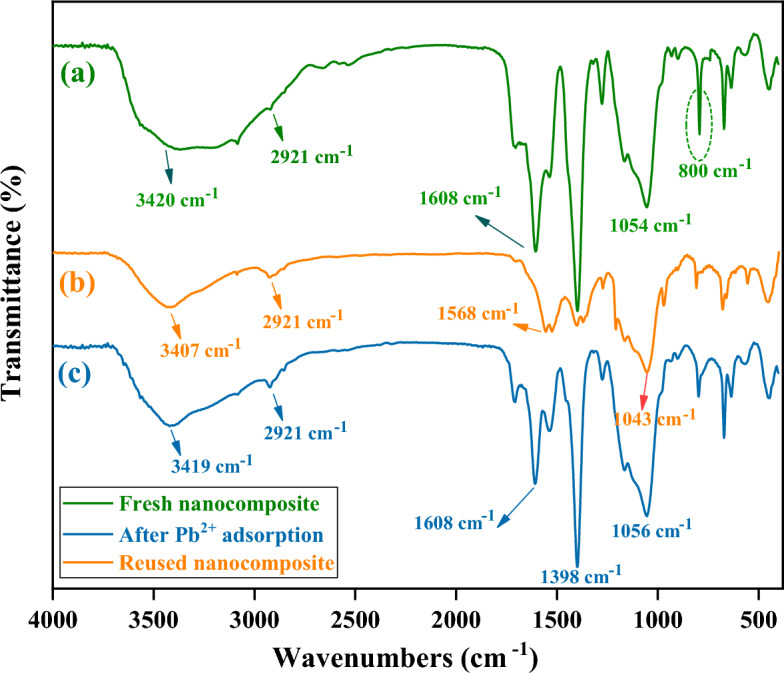
FTIR spectra of fresh DSS/MIL-88(A)-Fe composite (**a**), after pb2+ adsorption (**b**), and reused DSS/MIL-88(A)-Fe composite (**c**).

In order to further investigate the adsorption mechanism and the recycling capability of DSS/MIL-88(A)-Fe composite in the cyclic adsorption process for Pb^2+^ adsorption, the FT-IR spectra of DSS/MIL-88(A)-Fe composite before (Fig. 16a), after adsorption of Pb^2+^ (Fig. 16b), and also reused adsorbent after four catalytic runs (Fig. 16c) were analyzed.

As it is beheld from Fig. 16b, the characteristic peaks at 3420 and 1054 cm^−1^ attributed to vibrations of the DSS, shifted to 3407 and 1043 cm^−1^ and the intensity of the peaks weakened after the adsorption of Pb^2+^. Furthermore, the significant peaks at 1608 and 1398 cm^−1^ represented coordination between the carboxyl group and Fe^3+^, were red-shifted to 1568 and 1390 cm^−1^, and significantly weakened (Supplementary Information).

Also, from the FT-IR spectrum of reused adsorbent after four catalytic runs can be concluded that were matched in all the characteristic absorption bands such as shapes, positions, and frequencies with the FT-IR spectra of the fresh catalyst (Fig. 16c).

According to the data shown in Fig. 4f, the reused FE-SEM image of the DSS/MIL-88(A)-Fe composite asserts that not much change in the particle size or shape and morphology was observed after four catalytic runs.

A comparison of adsorption capacity and adsorption equilibrium time for Pb^2+^ by DSS/MIL-88(A)-Fe nanocomposite with some different adsorbents is listed in Table 5. Obviously, in previously reported works, despite the short adsorption equilibrium time of Pb^2+^, adsorbents demonstrated poor adsorption capacity compared to this work (Table 5, entries 1–6). Also, modified biochar showed a long adsorption time and relatively low adsorption capacity (Table 5, entry 7). In this study, the DSS/MIL-88(A)-Fe nanocomposite as an effective adsorbent of Pb^2+^ from an aqueous solution has high adsorption performance as well as a short adsorption time.

**Table 5:** Comparison of adsorption capacity of some reported Pb^2+^ adsorbents with DSS/MIL-88(A)-Fe composite.

Entry	Adsorbent	Adsorption capacity (mg/g)	Adsorption time (min)	Ref.
1	Chitosan immobilized on bentonite	15	45–60	^160^
2	Ni–P	39	20	^161^
3	Co–Fe_2_O_3_/Ni–Fe_2_O_3_	136	30	^162^
4	MIL-101 (Fe)/GO	128.6	15	^82^
5	SPS	83.66	5	^163^
6	HT NPs	169.5	10	^164^
7	Modified biochar	145	24 (h)	^165^
8	DSS/MIL-88(A)-Fe	220	45	This study

*SPS* sulfonated polystyrene, *HT NPs* gelatin-conjugated hematite nanoparticles.

## Conclusions

In summary, we synthesized a double-shelled periodic mesoporous organosilica nanospheres/MIL-88(A)-Fe nanocomposite in a conventional manner and comprehensively characterized through various techniques, including FTIR, XRD, BET, TEM, FE-SEM, EDX, and EDX-mapping analysis. Thanks to unique structure and remarkable properties of DSS/MIL-88A-Fe composite (such as an average size of 280 nm and 1.1 μm long attributed to the DSS and MOF, respectively, microporous structure and relatively large specific surface area (312.87 m^2^/g), which resulted from the coexistence of PMO and MOF, the composite described above exhibited excellent performance in the separation of Pb^2+^ from water with a maximum adsorption capacity of 230 mg/g with an effective adsorption rate of around 90 min. More importantly, one of the main advantages of this unprecedented composite is its cycling stability. To this end, the reusability of the DSS/MIL-88(A)-Fe was investigate, and the obtained results demonstrated that this adsorbent was preserved after 4 times regeneration, which illustrated its favorable performance in the removal of lead metal pollutants.

## Supplementary Information

Supplementary Information.

## Data Availability

The datasets used and/or analyzed during the current study are available from the corresponding author on reasonable request.
