# Supplementary material for: Synthesis of double-shelled periodic mesoporous organosilica nanospheres/MIL-88A-Fe composite and its elevated performance for Pb2+ removal in water
Source: Sci Rep. 2023 May 19;13:8092. doi: 10.1038/s41598-023-35149-w (PMC10199060; doi:10.1038/s41598-023-35149-w)
Supplement: Supplementary file 1 — Supplementary Information. [file 41598_2023_35149_MOESM1_ESM.docx]

**Supporting information**

**Synthesis of double-shelled periodic mesoporous organosilica nanospheres/MIL-88A-Fe composite and its elevated performance for Pb^2+^ removal in water**

Sara. S. E. Ghodsinia, Hossein Eshghi,* Arezu Mohamadi Nezhad

*Department of Chemistry, Faculty of Science, Ferdowsi University of Mashhad,*

*Mashhad 9177948974, Iran.*

*E-mail: heshghi@um.ac.ir*

**Experimental Section**

The FT-IR spectra were recorded on Thermo Nicolet Avatar 370 FT-IR. X-ray powder diffraction (XRD) was performed on a PANalytical Company X'Pert or MPD diffractometer with Cu Kα (λ = 0.154 nm) radiation. Nitrogen adsorption isothermswere measured on a Quantachrome Instruments version 2.2 using N_2_ as the adsorbate at -196 ˚C. Transmission electron microscopy (TEM) was carried out using an EM10C-100 KV microscope (ZEISS Company). FE-SEM images, EDX, and EDX-mapping were recorded by TESCAN (model: Sigma VP) scanning electron microscope operating at a low accelerating voltage, (ZEISS Company). Inductively coupled plasma optical emission spectroscopy (ICP-OES) was accomplished with a Varian Vista Pro CCD (Australia).

**Materials**

All reagents and chemicals were used without further purification. Absolute ethanol (EtOH, 99.9 %), concentrated ammonia (28 wt%), cetyltrimethylammonium bromide (CTAB,≥ 98 %), hydrochloric acid (HCl, 38%), tetraethyl orthosilicate (TEOS, 98%), 1,2-Bis(triethoxysilyl)ethane (BTEE, 97%), and Fumaric acid (HO₂CCH=CHCO₂H) used in this study were purchased from Sigma-Aldrich. Ferric chloride hexahydrate (FeCl_3_·6H_2_O) was bought from PubChem. Deionized water with a resistivity of 18.2 MΩ cm^-1^ was used in all experiments.

**Synthesis of double-shelled periodic mesoporous organosilica nanospheres**

Double-shelled PMO nanospheres were obtained via a sol-gel process based on literature reports [53]. In a typical synthesis, 0.16 g of CTAB was combined with a mixed solution of ethanol (30 mL), concentrated ammonia (1.0 mL), and deionized water (75 mL) at 40 °C for half-hour. Afterward, a mixture containing BTSE (0.119 g, 0.33 mmol) and TEOS (0.116 g, 0.56 mmol) was quickly added to the above mixture under vigorous stirring (1100 rpm) at 40 °C and kept for 24 h. To obtain two-layered mesostructured organosilica spheres, a mixture of TEOS and BTSE with an initial molar ratio was added to the mixture of the previous step. After further stirring for 24 h at 40 °C, the slurry was collected by centrifugation and washed with ethanol. The periodic mesostructured organosilica spheres were re-dispersed in 360 mL of deionized water and then transferred to a Teflon-lined stainless-steel autoclave, which was heated in an airflow electric oven at 140 °C for 5 h. After cooling the autoclave to room temperature, the product was collected by centrifugation. Subsequently, by the solvent-extraction process containing a solution containing 180 mL of ethanol and 360 µL of concentrated HCl, CTAB templates were removed from the product. Finally, double-shelled ethane-bridged PMO nanospheres were obtained after washing with ethanol three times and drying under a high vacuum at 80 °C overnight.

**Synthesis of MIL-88A-Fe**

To prepare the MIL-88A-Fe by the hydrothermal method, 2 mmol of FeCl_3_·6H_2_O (0.540 g) and 2 mmol of fumaric acid (0.232 g) were dispersed in 80 mL of ultra–pure water in the ultrasonic bath for 30 min, and then stirred for another 30 min. The resulting solution was transferred to a Teflon-lined autoclave (150 mL), and heated to 65°C for 12 h . The as-synthesized MIL-88A-Fe rods were obtained by centrifugation after cooling of Teflon-lined autoclave to room temperature. Finally, product was washed with deionized water several times, and dried under a vacuum at 65 °C.[59, 60]

**Synthesis of double-shelled ethane-bridged PMO nanospheres/MIL-88A-Fe composite (DSS/MIL-88(A)-Fe) composite**

As demonstrated in Scheme 1, the synthesis procedure of DSS/MIL-88(A)-Fe composite has proceeded through hydrothermal treatment. Firstly, a certain amount of double-shelled ethane-bridged PMO powder (10 wt%) was dispersed into 20 mL ultrapure water in a bath sonicator at room temperature for 1 h. Then, 2 mmol FeCl_3_·6H_2_O (0.540 g) and 2 mmol of fumaric acid (0.232 g) were added into 25 mL ultrapure water under continuous stirred for 30 min. Then, double-shelled ethane-bridged PMO solution was little by little dropped into the above solution and stirred forcefully for 2 h. Then the mixture was transferred into 100 mL autoclave, sealed and heated to 65 ^°^C for 12h. The final product was obtained as double-shelled ethane-bridged PMO/MIL-88A-Fe composite.

4.5. Adsorption experiments (preparation of Pb^2+^ ion solution)

***Adsorption process***

Various initial concentrations of Pb^2+^ solution (10, 20, 40, 60, 80, and 100 mg/L) were prepared by dissolving Pb(OAc)_2_ salt into deionized. Subsequently, DSS/MIL-88(A)-Fe composite (20 mg) was added in Pb^2+^ solution (50 mL) to adsorb Pb (II) at pH=6. The suspension was stirred during the test (5–150 min), and the temperature controlled by a thermostat water bath (25 ± 2 °C). The adsorption kinetic data were obtained by sampling 5 mL of suspension at different periods during the experiment's progress. The solid adsorbent composite was separated from the solution by filtration.

***Analytical methods***

The adsorption capacity (q_e_, mg.g^-1^) of Pb^2+^ by the solid adsorbent composite at equilibrium and as well as the removal efﬁciency (%) (R) of Pb^2+^ were calculated by the following formula: where C_0_ (mg/L) is the initial concentration of Pb (II) and C_e_ (mg/L) is the equilibrium concentration in the liquid phase. V is the volume of solution (mL), and m is the amount of the adsorbent (mg).

$$qe=\frac{\left( C0-Ce \right)\times V}{m} \left( 1 \right)$$

$$R= \frac{\left( C0-Ce \right)\times V}{C0} \times100\% (2)$$

Effect of time and initial contact concentration of Pb^2+^

Effect of time and initial contact concentration of Pb^2+^

Figure 1. Effect of contact initial concentration of Pb^2+^ and time on its adsorption.

Figure 2. Pb^2+^ adsorption isotherms for DSS, MIL-88A-Fe and DSS/MIL-88A-Fe composite at room

temperature with a concentration of 100 mg/L of Pb^2+^ solution.

Effect of adsorbent dosage

Figure 3. Effect of adsorbent dosage on adsorption capacity and removal efﬁciency of Pb^2+^.
